# Magnetic sphincter augmentation device removal: surgical technique and results at medium-term follow-up

**DOI:** 10.1007/s00423-021-02294-7

**Published:** 2021-08-30

**Authors:** Davide Bona, Greta Saino, Emanuele Mini, Francesca Lombardo, Valerio Panizzo, Marta Cavalli, Gianluca Bonitta, Giampiero Campanelli, Alberto Aiolfi

**Affiliations:** 1 Department of Biomedical Science for Health, Division of General Surgery, Istituto Clinico Sant’Ambrogio, University , of Milan, Via Luigi Giuseppe Faravelli, 16, 20149 Milan, Italy; 2 Department of Medicine and Surgery, Istituto Clinico Sant’Ambrogio, University of Insubria, Milan, Italy

**Keywords:** Gastroesophageal reflux disease (GERD), Magnetic sphincter augmentation device, Dysphagia, Removal

## Abstract

**Background:**

The magnetic sphincter augmentation (MSA) device has become a common option for the treatment of gastroesophageal reflux disease (GERD). Knowledge of MSA-related complications, indications for removal, and techniques are puzzled. With this study, we aimed to evaluate indications, techniques for removal, surgical approach, and outcomes with MSA removal.

**Methods:**

This is an observational singe-center study. Patients were followed up regularly with endoscopy, pH monitoring, and assessed for specific gastroesophageal reflux disease health-related quality of life (GERD-HRQL) and generic short-form 36 (SF-36) quality of life.

**Results:**

Five patients underwent MSA explant. Four patients were males and the median age was 47 years (range 44–55). Heartburn, epigastric/chest pain, and dysphagia were commonly reported. The median implant duration was 46 months (range 31–72). A laparoscopic approach was adopted in all patients. Intraoperative findings included normal anatomy (40%), herniation in the mediastinum (40%), and erosion (20%). The most common anti-reflux procedures were Dor (*n* = 2), Toupet (*n* = 2), and anterior partial fundoplication (*n* = 1). The median operative time was 145 min (range 60–185), and the median hospital length of stay was 4 days (range 3–6). The median postoperative follow-up was 41 months (range 12–51). At the last follow-up, 80% of patients were off PPI; the GERD-HRQL and SF-36 questionnaire were improved with DeMeester score and esophageal acid exposure normalization.

**Conclusion:**

The MSA device can be safely explanted through a single-stage laparoscopic procedure. Tailoring a fundoplication, according to preoperative patient symptoms and intraoperative findings, seems feasible and safe with a promising trend toward improved symptoms and quality of life.

**Supplementary Information:**

The online version contains supplementary material available at 10.1007/s00423-021-02294-7.

## Introduction

The magnetic sphincter augmentation (MSA), first introduced in 2007 and approved by FDA in 2012, has gained progressive acceptance as valuable therapeutic alternative for the surgical management of gastroesophageal reflux disease (GERD) [1, 2]. The MSA has been shown to be safe and effective with relief of reflux symptoms, discontinuation of daily proton pump inhibitors (PPIs), and reduction of esophageal acid exposure [3–6]. Compared to the traditional fundoplication, the MSA seems associated with less gas bloat symptoms with an increased ability to belch and vomit [7].

Concerns about possible complications and erosion of a foreign body placed at the gastroesophageal junction (GEJ) led to initial criticism because the past experience with the Angelchick and gastric banding devices [8, 9]. Previously published studies demonstrated that the rate of MSA removal ranges from 1.1 to 6.7% [11–13]. Principal reasons for MSA explant have been reported to be recurrent or persistent GERD, dysphagia, intractable chest/epigastric pain, or device erosion into the esophageal mucosa [13]. While the use of the MSA is increasing worldwide [14], studies focusing on device failure, techniques for removal, and outcomes remain essential in this stage of the adoption of the MSA device into surgical practice.

The purpose of this single-center cohort study was to analyze our experience with MSA removal and describe the surgical technique, intraoperative technical aspects, and medium-term follow-up results.

## Patients and methods

A retrospective, observational cohort study was designed and approved by the Institutional Review Board (ICSA-IRA#2020–106). The prospectively collected database at our tertiary care University hospital was queried to identify all adult patients (≥ 18-year-old) that underwent MSA device removal from November 2015 to January 2021. The study was conducted according to the principles of Helsinki declaration and informed consent obtained from all included patients. Patients’ characteristics, timing from the index operation, operative variables, and postoperative course were analyzed. Preoperative evaluation included chest X-ray with bead count, barium swallow study, and upper endoscopy. High resolution manometry and 24 h pH monitoring were performed preoperatively in patients with no evidence of device erosion. Chest computer tomography was performed in one patient with device erosion.

A gastrografin swallow study was performed on postoperative day 1. If negative patients were allowed to resume a semiliquid diet, follow-up visits were scheduled 2 months after discharge and then every 6 months after surgery. Barium swallow study and upper endoscopy were performed for investigational purposes every year after the operation or in case of recurrent symptoms. An ambulatory pH impedance study was performed yearly after the procedure. Disease-specific gastroesophageal reflux disease health-related quality of life (GERD-HRQL) and generic short-form 36 (SF-36) questionnaires were administered before surgery and during follow-up [15–17].

### Surgical technique

**Fig. 1:**
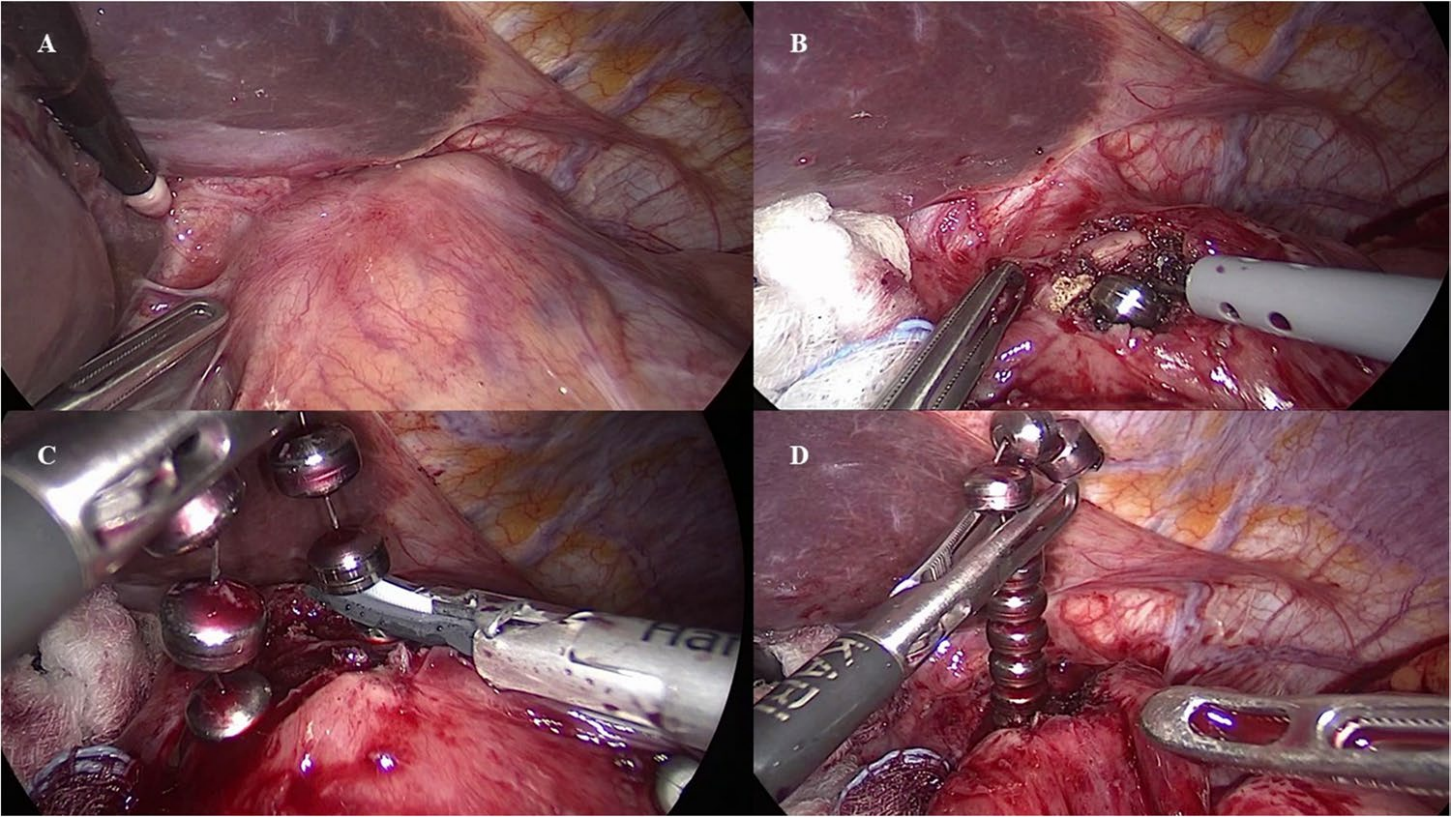
**A–D** The scar tissue at the gastroesophageal junction corresponding to the underneath MSA device is identified (**A**). A monopolar electrocautery hook is used to open the fibrotic scar tissue to expose at least a couple of titanium beads (**B**). The titanium wire, connecting the independent sphincter beads, is sectioned with an ultrasonic scalpel (**C**). The MSA may be removed entirely or in two pieces (**D**)

The patient is positioned supine and in reverse Trendelenburg position. The surgeon stands within patient legs. Pneumoperitoneum is established with a Veress needle, and the abdomen is entered through the prior port-sites. Adhesions and scar tissue between the stomach, the left lobe of the liver, and the diaphragm are divided to expose the anterior aspect of the GEJ. The scar tissue at the GEJ corresponding to the underneath MSA device is identified (Fig. 1A). A monopolar electrocautery hook is used to open the fibrotic scar tissue for about 1 cm to expose at least a couple of anterior titanium beads (Fig. 1B). The incision may be enlarged *à la demande* laterally to expose more titanium beds. The harmonic scalpel is then used to cut the titanium wire, while in some cases, the MSA device can be opened by opening the clasp with an anticlockwise movement (Fig. 1C). While this scalpel is not designed to be used in contact with metal or plastic instruments or objects, this off-label technique has been the only reported strategy to cut the MSA titanium wire. Therefore, caution should be paid to avoid thermal visceral injuries in this phase. The sphincter may be removed entirely or in two pieces (Fig. 1D). The total bead count in the explanted device should be confirmed, while the device is removed. Concomitant intraoperative endoscopy is used to identify the GEJ and to check the integrity of the esophageal mucosa. An anterior Dor or posterior Toupet fundoplication with crural repair are performed depending on preparative patient symptoms and intraoperative findings. A low-pressure Dor fundoplication is preferred in patients with predominant dysphagia and chest pain, while in case of heartburn, evidence of hiatus hernia, or recurrent GERD at the preoperative pH study with pathologic esophageal acid exposure, a posterior Toupet fundoplication with crural repair is performed. Finally, in case of MSA erosion, an anterior partial plication is fashioned and used as a patch to cover and protect the fibrotic capsule incision in attempt to reduce the risk of esophageal leak development (Supplementary Video).

### Statistical analysis

The Statistical Package for Social Science (SPSS, Version 23, SPSS, Inc., Chicago, IL) was used for data analysis [18]. Quantitative variables were expressed as median and range. Fisher’s exact test was performed where appropriate. A *p* < 0.05 was considered significant.

## Results

During the study period, 32 MSA devices were implanted at our institution and five patients underwent MSA explant. One patient (3.1%) was from our center, while the other four were referred from other institutions. Demographic and clinical characteristics of the patients’ population are presented in Table 1. Four patients were males and the median age was 47 years (range 44–55). The median body mass index (BMI) was 23.1 kg/m^2^ (range 20.9–24.3). Reasons for MSA explant were recurrent heartburn (*n* = 3), epigastric/chest pain (*n* = 3), dysphagia non-responding to esophageal dilation (*n* = 2), regurgitation (*n* = 2), and device erosion (*n* = 1). The median implant duration was 46 months (range 31–72). High resolution esophageal manometry and 24-h pH impedance monitoring were performed in four patients with no evidence of underlying motility or lower esophageal sphincter disorders. Compared with preoperative data, the 24-h pH impedance study was improved but still pathologic in the two patients complaining heartburn and regurgitation. In one of these patients, preoperative endoscopy shows grade A esophagitis with 2 cm hiatus hernia. The preoperative gastrografin swallow study shows a delayed esophageal empting without esophageal dilation in one patient. One patient underwent device removal for mucosal erosion 31 months after MSA implant. The patient complained intermitted dysphagia and chest pain. The upper endoscopy showed the presence of erosion with one beds migrated into the esophageal lumen.

**Table 1:** Demographic and clinical characteristics of the patient population

ID	Age	Sex	Symptoms	Endoscopy	HRM		DeMeester score	Preop GERD-HRQL	Size (no. bead)	Year of implant	Time to explant (mos)	OR findings	Surgical procedure	OT (min)	HLOS (day)	Follow-up				
					EP	LES (mmHg)										mos	GERD-HRQL	DeMeester score	Dysphagia	PPI
1	44	M	H, CP	Normal	Normal	31	10.8	26	12	2012	66	Normal anatomy	DF	105	3	41	6	9.5	None	N
2*	52	F	D, CP	Erosion	-	-	-	17	15	2017	31	Erosion	APF	60	6	12	4	8.4	None	Y
3	44	M	D	Normal	Normal	28	12.4	14	13	2011	72	Normal anatomy	DF	185	4	48	6	11.8	None	N
4	55	M	H, EP, R	HH	Normal	21	40.8	38	14	2014	25	HH	TF + CR	145	5	51	9	10.9	Occasional	N
5	47	M	H, R	HH; esophagitis	Normal	19	52.6	24	13	2013	46	HH	TF + CR	155	3	37	5	13.2	None	N

*M* male, *F* female, *H* heartburn, *CP* chest pain, *D* dysphagia, *EP* epigastric pain, *R* regurgitation, *HH* hiatus hernia, *HRM* high resolution manometry, *EP* esophageal peristalsis, *LES* lower esophageal sphincter, *GERD-HRQL* gastroesophageal reflux disease health-related quality of life, *Mos* months, *OR* operative room, *DF* Dor fundoplication, *APF* anterior partial fundoplication, *TF* Toupet fundoplication, *CR* crural repair, *OT* operative time, *Min* minutes, *HLOS* hospital length of stay, *PPI* proton pump inhibitors. *From our center, presented in the Supplementary Video

All patients underwent a single-stage laparoscopic removal with intraoperative endoscopic assistance. Significant operative findings included normal anatomy (40%), herniation in the mediastinum (40%), and erosion (20%). Hiatus hernia was found and repaired in two patients (40%). The most common anti-reflux procedure performed in conjunction with MSA device removal was an anterior Dor fundoplication (DF) (*n* = 2) followed by Toupet fundoplication (TF) and crural repair (*n* = 2). In the patient that experienced MSA erosion, an anterior partial fundoplication was performed (*n* = 1) after removal. The median operative time was 145 min (range 60–185), and the median hospital length of stay was 4 days (range 3–6). There were no intraoperative complications, the intraoperative blood loss was negligible, and none of the patients required postoperative transfusion. None of the patients experienced esophageal leak. There was no morbidity related to the procedures nor mortality.

**Fig. 2:**
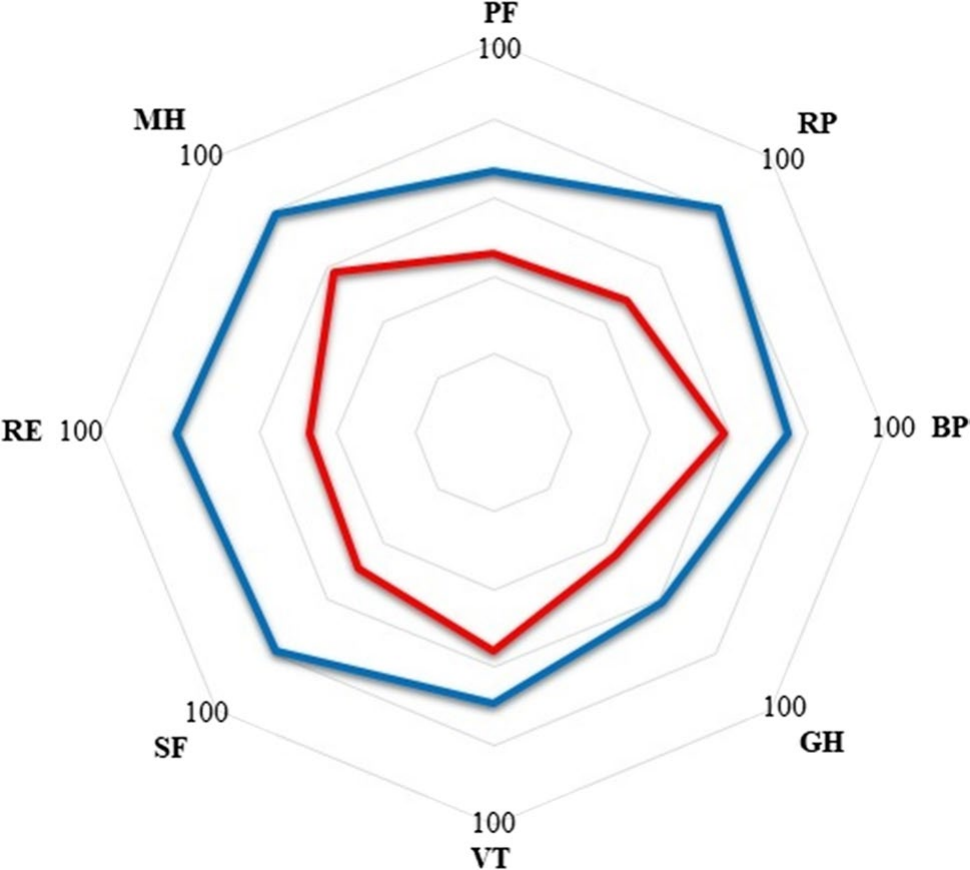
Preoperative and postoperative quality of life according to the 36-item short-form health survey health questionnaire. Each item is evaluated on a 0 to 100 scale. FC, functional capacity; PA, physical aspect; BP, bodily pain; GH, general health; VT, vitality; SF, social function; RE, role emotional; MH, mental health

The median postoperative follow-up was 41 months (range 12–51). At the last follow-up, the median GERD-HRQL was significantly improved compared to preoperative data (6 ± 1.8 vs. 24 ± 9.3; *p* < 0.05), 80% of patients were off PPI, and patients’ quality of life assessed with the SF-36 questionnaire was improved (Fig. 2).

## Discussion

This study shows that MSA device can be safely explanted through a single-stage laparoscopic procedure. An anterior Dor or Toupet fundoplication can be safely performed in case of dysphagia/chest pain or recurrent GERD, respectively, while in case erosion, an anterior partial plication may be beneficial to prevent the development of esophageal leak.

After its introduction into clinical practice, the MSA device has progressively gained popularity and emerged as a valuable therapeutic alternative to fundoplication in patients with GERD [19, 20]. Several single-arm trials have established consistent and long-term improvement of GERD symptoms scores, esophageal acid exposure, quality of life, and decreased use of PPI [2–6, 21, 22]. Given the purpose of the MSA and its conceptual similarity to the Angelchik device, there have been concerns about the possibility of complications and esophageal erosion. Previous studies reported MSA removal because recurrent GERD symptoms, dysphagia, and erosion [10–12, 23–25]. The incidence of device removal in the literature ranges from 1.1 to 6.7% [10–12]. Specifically, a recent safety analysis of the first 1000 MSA reported a reoperation and removal rate of 3.4% because postoperative dysphagia non-responding to endoscopic dilation, recurrent GERD symptoms, and mucosal erosion [11]. Another study by Smith et al. stated an overall removal rate of 2.7% because of dysphagia (1.6%), GERD (0.6%), and erosion (0.15%) up to 1.4 years mean follow-up [12]. Other authors described a removal rate of 6.7%, most commonly for reflux and dysphagia [10]. It has been shown that different factors seem associated with an increased risk of device removal such as device undersizing, minimal hiatal dissection, and patient-specific risk factors (i.e., steroid use, poorly controlled diabetes) [14].

In case of device erosion, different solutions for MSA removal have been described ranging from single-stage endoscopic retrieval to hybrid endo-laparoscopic two-stage strategies [26–29]. In case of dysphagia or recurrent GERD symptoms, a single-stage laparoscopic approach under endoscopic assistance has been reported [10, 30]. In our experience, the most challenging operative phase is the mobilization and extraction of the posterior part of the device that may result strongly adherent to the posterior esophageal wall. Any attempt to avoid iatrogenic mucosal injuries related to inadvertent device tractions should be made. Therefore, we prefer to mobilize the device laterally on the left or right side in order to expose more possible beads. Finally, the wire is sectioned, and the remaining posterior part of the device is extracted contralateral.

After MSA removal, different surgical strategies have been reported with Dor fundoplication, Toupet fundoplication, Nissen fundoplication, reconstruction of the His angle, MSA reimplantation with hiatal repair, or no additional anti-reflux procedures [10, 13, 14]. However, a robust evidence-based indication on the superiority of one approach over another is still lacking, and a precise treatment algorithm is still to be defined after MSA removal. In our experience, the choice of the most suitable anti-reflux procedure was based on preoperative patients’ symptoms, esophageal acid exposure on pH monitoring, and/or residual anatomy after explant. Specifically, a low-pressure Dor fundoplication with right crus exposure and minimal esophageal mobilization was preferred in patients with predominant dysphagia and chest pain. In these patients, the His angle is reconstructed by suturing the fundus to the left side of the esophagus and left crus. The fundus is then rolled over the top of the esophagus and sutured to the hiatus and right crus. Differently, in case of heartburn, hiatus hernia, recurrent pathologic GERD assessed with preoperative pH study, or intraoperative evidence or hiatus hernia a posterior Toupet fundoplication with crural dissection and hiatus reapproximation was performed. Finally, in case of erosion, an anterior partial plication with minimal GEJ dissection was preferred. The gastric fundus was used to create an anterior patch to cover anteriorly the fibrotic capsule incision in attempt to reduce the risk of esophageal leak. In these cases, we believe that complete esophageal dissection is unnecessary and dangerous because the inflammatory fibrotic reaction at the GEJ enhanced by the device erosion.

Medium-term outcomes assessed with 24-h pH study after MSA removal showed normalization of the DeMeester score and esophageal acid exposure. This effect may be theoretically related to both the presence of a fibrotic reaction around the GEJ, as described by Tatum et al. [13], in conjunction with fashioning a “patient-tailored” fundoplication according to preoperative symptoms and intraoperative findings. A promising trend toward improved symptoms, quality of life, patients’ satisfaction, and PPI suspension was noticed in our patient cohort. Therefore, “tailoring” a fundoplication according to preoperative symptoms, esophageal acid exposure, and/or residual anatomy after explant seems promising in improving symptoms, quality of life, and esophageal acid exposure in the medium-term follow-up. Limitations of the study are the retrospective designs, the limited and heterogeneous patient population, and narrow follow-up. Therefore, caution should be paid while interpreting our results. Future multicenter studies are warranted to deeply assess which is the most suitable surgical strategy to adopt in case MSA should be removed.

## Conclusion

The MSA device can be safely explanted through a single-stage laparoscopic procedure. Tailoring a fundoplication, according to preoperative patient symptoms and intraoperative findings, seems feasible and safe with a promising trend toward improved esophageal acid exposure, symptoms, and quality of life in the medium term follow-up.

## Supplementary Information

Below is the link to the electronic supplementary material.

Supplementary file1 (MP4 258814 KB)

## References

[1] Ganz RA, Peters JH, Horgan S, Bemelman WA, Dunst CM, Edmundowicz SA (2013). Esophageal sphincter device for gastroesophageal reflux disease. N Engl J Med.

[2] Reynolds JL, Zehetner J, Bildzukewicz N, Katkhouda N, Dandekar G, Lipham JC (2014). Magnetic sphincter augmentation with the LINX device for gastroesophageal reflux disease after U.S. Food and Drug Administration approval. Am Surg.

[3] Bonavina L, Saino GI, Bona D, Lipham J, Ganz RA, Dunn D, DeMeester T (2008). Magnetic augmentation of the lower esophageal sphincter: results of a feasibility clinical trial. J Gastrointest Surg.

[4] Ganz RA, Edmundowicz SA, Taiganides PA, Lipham JC, Smith CD, DeVault KR (2016). Long-term outcomes of patients receiving a magnetic sphincter augmentation device for gastroesophageal reflux. Clin Gastroenterol Hepatol.

[5] Bonavina L, Saino G, Bona D, Sironi A, Lazzari V (2013). One hundred consecutive patients treated with magnetic sphincter augmentation for gastroesophageal reflux disease: 6 years of clinical experience from a single center. J Am Coll Surg.

[6] Bonavina L, DeMeester T, Fockens P, Dunn D, Saino G, Bona D, Lipham J, Bemelman W, Ganz RA (2010). Laparoscopic sphincter augmentation device eliminates reflux symptoms and normalizes esophageal acid exposure: one- and 2-year results of a feasibility trial. Ann Surg.

[7] Aiolfi A, Asti E, Bernardi D, Bonitta G, Rausa E, Siboni S, Bonavina L (2018). Early results of magnetic sphincter augmentation versus fundoplication for gastroesophageal reflux disease: Systematic review and meta-analysis. Int J Surg.

[8] Pence MM, Hubbard M, Singla MB, Young PE (2015). Esophagogastric fistula caused by an Angelchik antireflux prosthesis. ACG Case Rep J.

[9] Spann MD, Aher CV, English WJ, Williams DB (2017). Endoscopic management of erosion after banded bariatric procedures. Surg Obes Relat Dis.

[10] Asti E, Siboni S, Lazzari V, Bonitta G, Sironi A, Bonavina L (2017). Removal of the magnetic sphincter augmentation device: surgical technique and results of a single-center cohort study. Ann Surg.

[11] Lipham JC, Taiganides PA, Louie BE, Ganz RA, DeMeester TR (2015). Safety analysis of first 1000 patients treated with magnetic sphincter augmentation for gastroesophageal reflux disease. Dis Esophagus.

[12] Smith CD, Ganz RA, Lipham JC, Bell RC, Rattner DW (2017). Lower esophageal sphincter augmentation for gastroesophageal reflux disease: the safety of a modern implant. J Laparoendosc Adv Surg Tech A.

[13] Tatum JM, Alicuben E, Bildzukewicz N, Samakar K, Houghton CC, Lipham JC (2019). Removing the magnetic sphincter augmentation device: operative management and outcomes. Surg Endosc.

[14] Alicuben ET, Bell RCW, Jobe BA, Buckley FP, Daniel Smith C, Graybeal CJ, Lipham JC (2018). Worldwide experience with erosion of the magnetic sphincter augmentation device. J Gastrointest Surg.

[15] Velanovich V (1998). Comparison of generic (SF-36) vs. disease-specific (GERD-HRQL) quality-of-life scales for gastroesophageal reflux disease. J Gastrointest Surg.

[16] Ware JE, Sherbourne CD (1992). The MOS 36-item short-form health survey (SF-36). I. Conceptual framework and item selection. Med Care.

[17] Porta A, Aiolfi A, Musolino C, Antonini I, Zappa MA (2017) Prospective comparison and quality of life for single-incision and conventional laparoscopic sleeve gastrectomy in a series of morbidly obese patients. Obes Surg 27(3):681–687. 10.1007/s11695-016-2338-2 10.1007/s11695-016-2338-2 27686234

[18] R Development Core Team (2015) A language and environment for statistical computing. R Foundation for Statistical Computing, Vienna, Austria. ISBN 3-900051-07-0

[19] Schwameis K, Ayazi S, Zheng P, Grubic AD, Salvitti M, Hoppo T, Jobe BA (2021). Efficacy of magnetic sphincter augmentation across the spectrum of GERD disease severity. J Am Coll Surg.

[20] Asti E, Aiolfi A, Lazzari V, Sironi A, Porta M, Bonavina L (2018) Magnetic sphincter augmentation for gastroesophageal reflux disease: review of clinical studies. Updates Surg 70(3):323–330. 10.1007/s13304-018-0569-6 10.1007/s13304-018-0569-6 30022361

[21] Lipham JC, DeMeester TR, Ganz RA, Bonavina L, Saino G, Dunn DH, Fockens P, Bemelman W (2012). The LINX® reflux management system: confirmed safety and efficacy now at 4 years. Surg Endosc.

[22] Saino G, Bonavina L, Lipham JC, Dunn D, Ganz RA (2015). Magnetic sphincter augmentation for gastroesophageal reflux at 5 years: final results of a pilot study show long-term acid reduction and symptom improvement. J Laparoendosc Adv Surg Tech A.

[23] Bonavina L, DeMeester TR, Ganz RA (2012) LINX(™) Reflux management system: magnetic sphincter augmentation in the treatment of gastroesophageal reflux disease. Expert Rev Gastroenterol Hepatol 6(6):667–74 10.1586/egh.12.47 23237251

[24] Ayazi S, Zheng P, Zaidi AH, Chovanec K, Chowdhury N, Salvitti M, Komatsu Y, Omstead AN, Hoppo T, Jobe BA (2020). Magnetic sphincter augmentation and postoperative dysphagia: characterization, clinical risk factors, and management. J Gastrointest Surg.

[25] DeMarchi J, Schwiers M, Soberman M, Tokarski A (2021) Evolution of a novel technology for gastroesophageal reflux disease: a safety perspective of magnetic sphincter augmentation. Dis Esophagus doab036 10.1093/dote/doab036 PMC8597906 34117494

[26] Yeung BPM, Fullarton G (2017). Endoscopic removal of an eroded magnetic sphincter augmentation device. Endoscopy.

[27] Bauer M, Meining A, Kranzfelder M, Jell A, Schirren R, Wilhelm D, Friess H, Feussner H (2015). Endoluminal perforation of a magnetic antireflux device. Surg Endosc.

[28] Parmar AD, Tessler RA, Chang HY, Svahn JD (2017). Two-stage explantation of a magnetic lower esophageal sphincter augmentation device due to esophageal erosion. J Laparoendosc Adv Surg Tech A.

[29] Salvador R, Costantini M, Capovilla G, Polese L, Merigliano S (2017). Esophageal penetration of the magnetic sphincter augmentation device: history repeats itself. J Laparoendosc Adv Surg Tech A.

[30] Stetler JL, Gill S, Patel A, Davis SS, Lin E (2015). Surgical technique for laparoscopic removal of a magnetic lower esophageal sphincter augmentation device. J Laparoendosc Adv Surg Tech A.

